# Endometriosis and Reproductive Sparing Surgery: A Narrative Review and AGREE II-S-Based Evaluation of International Guidelines

**DOI:** 10.3390/jcm15010380

**Published:** 2026-01-04

**Authors:** Giovanni Pecorella, Andrea Morciano, Radmila Sparic, Gernot Hudelist, Ertan Saridogan, Marta Stojković, Andrea Tinelli

**Affiliations:** 1 Department of Obstetrics and Gynecology, and CERICSAL (CEntro di RIcerca Clinico SALentino), “Veris delli Ponti Hospital”, 73020 Scorrano, Italyandreatinelli@gmail.com (A.T.); 2 Department of Gynaecology and Obstetrics, Pia Fondazione “Card. G. Panico”, 73039 Tricase, Italy; 3 School of Medicine, University of Belgrade, 11000 Belgrade, Serbiamstojkovic037@gmail.com (M.S.); 4 Clinic of Gynecology and Obstetrics, University Clinical Centre of Serbia, 11000 Belgrade, Serbia; 5 Department of Gynecology, Center for Endometriosis, Hospital St. John of God, 1020 Vienna, Austria; 6 Rudolfinerhaus Private Clinic & Campus, 1190 Vienna, Austria; 7 Department of Gynecology and Oncology, Jagiellonian University, 31-007 Krakow, Poland; 8 Department of Gynaecology, University College London Hospitals NHS Foundation Trust, London NW1 2PG, UK; 9 EGA Institute for Women’s Health, University College London Hospital NHS Foundation Trust, London NW1 2PG, UK

**Keywords:** endometriosis guidelines, minimally invasive surgery, surgical treatment, quality of life, patient-reported outcomes, AGREE II, ESHRE, S2k, ACOG, NICE

## Abstract

Endometriosis is a complex disease that may affect a woman’s fertility and quality of life. Owing to substantial variations in symptom severity, lesion distribution, and reproductive impact, its management presents considerable clinical challenges. The most recent internationally recognized guidelines include those issued by the European Society of Human Reproduction and Embryology (ESHRE, 2022), the German Society of Gynecology and Obstetrics (DGGG/SGGG/OEGGG S2k, 2025), the World Endometriosis Society (WES), the National Institute for Health and Care Excellence (NICE, 2024), and the American College of Obstetricians and Gynecologists (ACOG, reaffirmed 2022). To provide a comprehensive overview of these recommendations, we critically compared these guidelines, with particular emphasis on the recently updated German S2k guideline. Searches were conducted through PubMed and institutional repositories using selected key terms, and the AGREE II tool (Appraisal of Guidelines for Research and Evaluation) was employed to assess methodological quality. Key clinical domains examined included indications for conservative and radical surgery, management of endometriomas and deep infiltrating endometriosis (DIE), the role of surgery before Assisted Reproductive Technology (ART), the impact of second-look procedures, and integration of psychosocial dimensions via Patient-Reported Outcome Measures (PROMs). The results show a general trend toward interdisciplinary treatment models, cautious use of radical resection techniques, and customized, symptom-based surgical interventions. Despite ongoing disagreements, there is general agreement on collaborative decision-making, preserving fertility, and adjusting surgery time and technique between the guidelines to meet the requirements of individual patients.

## 1. Introduction

Endometriosis is a chronic inflammatory disease, characterized by the presence of endometrial tissue outside the uterine cavity [1]. Endometriotic lesions are most seen within the pelvic cavity or pelvic organs, although instances of extra-pelvic involvement have been documented, affecting distant areas such as the pleura, pericardium, and central nervous system [2,3,4]. The classical clinic tetrad is represented by dysmenorrhea [5], dyspareunia [6], dysuria [7], and dyschezia [8]. Many of the endometriosis classifications that are now in use are probably didactic and center on the depth/size and location of lesions, along with adhesions. One of the first archetypes was the 1979 revision of the American Society for Reproductive Medicine (rASRM) categorization [9,10], later complemented by more detailed systems such as the #Enzian classification, which focuses on deep endometriosis, and the AAGL score, designed to assess surgical complexity [11,12]. While several classification systems have been proposed, their primary role remains descriptive, and their direct impact on surgical decision-making is still limited. Although the severity of symptoms does not always correlate with the severity of endometriosis, extent of the condition remains important when surgery is considered, as this would determine the difficulty or complexity of surgery. Hence, there are ongoing attempts to fine-tune the existing classification systems or produce new ones. It is often acknowledged that one of the most difficult gynecological treatments is endometriosis surgery [13].

There are many treatment options accessible today, ranging from prescription medications to dietary and lifestyle changes to progressively extensive surgical procedures [14]. The EndoCost study, conducted across 12 reference centers in 10 European countries, estimated the societal cost of endometriosis, including healthcare expenses, transportation, and productivity loss. The average annual cost per patient exceeded EUR 9500, with 80% attributed to reduced work productivity [15]. This demonstrates that the chronic nature of endometriosis and the associated pain both drive the need for an effective treatment that can control its impact in the long term. Surgery represents a valuable treatment option for patients with endometriosis [16]. Surgery should ideally be globally standardized and be accessible to those both in highly developed and rural settings, to ensure equitable and effective care. To achieve this challenging goal, clinical guidelines are essential. Surgical management was deliberately selected as the focus of this review because it represents the area of greatest heterogeneity among guidelines and carries the highest potential impact on fertility, organ function, and long-term quality of life.

## 2. Materials and Methods

This manuscript is designed as a narrative review combined with an AGREE II-S-based appraisal of international clinical practice guidelines on the surgical management of endometriosis. We performed a comprehensive literature review using the Scopus and PubMed databases, covering the period from April 2000 to April 2025, to gather current evidence on the optimal surgical management of endometriosis. These databases were selected because they comprehensively cover peer-reviewed biomedical and surgical literature; additional databases were considered but were not expected to yield substantially different guideline documents. Earlier documents were also considered if they remained current through reaffirmation or official updates (e.g., ACOG 2010, reaffirmed 2022). Our analysis focused on identifying and appraising international clinical practice guidelines on endometriosis. The literature search aimed to retrieve documents issued by recognized professional societies or health authorities that provide diagnostic or therapeutic recommendations for endometriosis management. The search strategy employed a combination of keywords, including “endometriosis,” “guidelines,” “recommendations,” “consensus,” “management,” and the names of major societies (ESHRE, NICE, ACOG, WES, SEUD, and DGGG/SGGG/OEGGG S2k). Only the most recent, peer-reviewed, and officially published guideline documents available in English between 2010 and 2025 were included for comparison and critical evaluation. Additional relevant sources were identified through manual cross-referencing of cited literature. Studies primarily focused on hysterectomy were excluded, as this procedure represents a definitive, non–fertility-sparing strategy and falls outside the scope of conservative surgical management addressed in this review. The review primarily focused on surgical techniques for endometriosis management, comparing the traditional approach—often involving definitive surgery such as hysterectomy—with more conservative, fertility-sparing interventions. Although the age range reported across the included studies was wide, the majority of patients were of reproductive age. We identified six international guidelines addressing surgical treatment for endometriosis, the contents of which are compared in detail. The analyzed guidelines include the updated European Society of Human Reproduction and Embryology (ESHRE) guideline (2022), the German S2k guideline (2025), NICE (2024), WES (2013), SEUD (2023), and ACOG (2010, reaffirmed 2022). Furthermore, we evaluated each guideline using the AGREE II-S instrument, assessing their methodological rigor, clinical applicability, and potential for implementation in daily practice. The functioning and evaluation criteria of the AGREE II-S tool are discussed in the following section. The review primarily focused on comparing the surgical recommendations provided by international guidelines for the management of endometriosis. Specifically, we analyzed how each guideline addresses surgical options, contrasting traditional approaches—often involving definitive surgery such as hysterectomy—with more conservative, fertility-sparing strategies. The target population considered across these guidelines includes women of reproductive and premenopausal age. We identified six international guidelines addressing the surgical management of endometriosis: ESHRE 2022, S2k 2025 (German guidelines), NICE 2024, WES 2013, SEUD 2023, and ACOG 2010 (reaffirmed 2022). Each document was appraised using the AGREE II-S instrument to evaluate methodological rigor, clinical applicability, and potential for implementation in daily practice. The functioning and evaluation criteria of the AGREE II-S tool are described in the following section.

### 2.1. Evaluation Framework and Scoring Methodology of AGREE II and AGREE II-S Tools for Guideline Appraisal

The methodological quality of each included guideline was independently assessed using the Appraisal of Guidelines for Research and Evaluation II—Surgical adaptation (AGREE II-S) instrument, a standardized framework designed to appraise the methodological rigor and transparency of clinical practice guidelines. The appraisal was conducted by two independent reviewers, both gynecologic surgeons with expertise in minimally invasive surgery and guideline evaluation. Formal inter-rater reliability statistics were not calculated, in line with AGREE II-S methodology, as the tool is primarily intended for structured qualitative appraisal rather than quantitative agreement analysis. Prior to assessment, both reviewers completed standardized AGREE II-S training to ensure consistent interpretation of all items. The AGREE II-S tool evaluates six domains: (1) Scope and Purpose, (2) Stakeholder Involvement, (3) Rigor of Development, (4) Clarity of Presentation, (5) Applicability, and (6) Editorial Independence. Compared with the original AGREE II framework, the surgical adaptation (AGREE II-S) expands its analytical sensitivity by incorporating aspects such as implementation feasibility, equity in healthcare access, and engagement of end-users during development. Each of the 23 items was rated on a 7-point Likert scale (1 = strongly disagree; 7 = strongly agree). Domain scores were calculated as percentages of the maximum possible value, with an overall composite score expressed as a proportion of the theoretical maximum (161 points). Discrepancies greater than one point between reviewers were first discussed to reach consensus; if disagreement persisted, a third senior reviewer was consulted. Final scores were used to compare the methodological robustness and practical applicability of the analyzed guidelines and to contextualize observed variations in their clinical recommendations [17]. AGREE II-S scores were used to support comparative interpretation of guideline quality and transparency, and did not directly weight or determine the clinical conclusions discussed in the manuscript. The specific ratings according to the AGREE II-S criteria are presented in Table 1.

### 2.2. Surgical Management of Ovarian Endometriomas: Indications, Techniques, and Reproductive Considerations

The surgical approach to ovarian endometriomas remains one of the most debated aspects in the treatment of endometriosis, particularly when balancing the goals of pain relief, preservation of ovarian function, and optimization of fertility outcomes [18]. Across recent international guidelines, there is broad agreement that surgery should be considered only when clinically indicated, typically in cases of significant pain, infertility, or when imaging raises concern for malignancy [19]. Both the ESHRE 2022 and S2k 2025 guidelines explicitly state that endometriomas should not be excised solely based on their presence, particularly in asymptomatic women or those planning assisted reproductive technologies (ART) [20,21]. Similarly, the WES 2021 consensus reinforces this conservative approach, emphasizing individualized assessment rather than automatic surgical intervention [22]. None of the reviewed guidelines provides a universally accepted size threshold for intervention in ovarian endometriomas. The European Society of Human Reproduction and Embryology (ESHRE) 2022 guideline explicitly states that the decision for surgery should not be based solely on cyst diameter, but rather on symptoms, suspicion of malignancy or access issues for oocyte retrieval in ART-settings. National Institute for Health and Care Excellence (NICE) 2024 refers to cyst size as an adjunct for follow-up or specialist referral in some cases, but likewise stops short of defining a mandatory cut-off. The NICE 2024 guideline similarly advises specialist referral and follow-up for women with persistent or complex ovarian cysts, particularly when endometriomas are suspected or fertility is affected. SEUD 2023 adds that ovarian surgery should be avoided in patients with adequate ovarian function and stable cysts, reinforcing a conservative trend [23,24]. They emphasize the importance of relevant symptoms or the presence of cysts > 3 cm as reasonable arguments for surgical consideration. Importantly, this reference to cyst size reflects a contextual clinical consideration rather than a formal or mandatory surgical threshold [20,21,22]. Neither ESHRE nor S2k provide an explicit size cut-off for surgical intervention. Both guidelines emphasize that surgery should be considered based on clinical symptoms, suspicion of malignancy, or technical difficulties during oocyte retrieval, rather than cyst diameter alone, particularly in women undergoing fertility evaluation or treatment [20]. Conversely, NICE 2024 avoids any fixed dimensional criteria, stating instead that surgery should be limited to cases with significant clinical symptoms or suspicion of malignancy. For subfertile women, NICE specifically advises against performing surgery solely to improve fertility outcomes, recommending instead referral to a fertility specialist for individualized management [23]. ACOG, too, remains noncommittal on a size threshold and instead centers the decision on symptom severity or poor response to medical treatment [5,25]. A particularly nuanced point comes from ESHRE, which notes that the presence of an endometrioma—even of considerable size—does not necessarily equate to clinical relevance. In the absence of pain or impact on ovulation, it may be safely observed. This introduces the idea that not all endometriomas need to be removed, especially smaller, asymptomatic ones—an important shift in modern practice that only some guidelines explicitly address [26,27]. The results of this comparison are shown in Table 2.

#### 2.2.1. Surgical Techniques: Cystectomy Versus Drainage/Ablation

There is broad consensus that laparoscopic cystectomy provides superior long-term outcomes in terms of pain relief and recurrence compared to drainage or ablation [28]. This is confirmed by ESGE/ESHRE/WES joint recommendations [29], as well as by ESHRE 2022, and S2k, which all favor cystectomy as the gold standard for managing symptomatic endometriomas [30]. However, a major point of divergence arises in women with diminished ovarian reserve or fertility issues. Both ESHRE 2022 and S2k 2025 introduce the concept of technique modulation, suggesting that ablative techniques (e.g., laser vaporization or plasma energy) may be appropriate alternatives when there is concern about follicular damage [31,32]. In particular, ablative techniques are considered in cases of bilateral disease, diminished ovarian reserve, or when cystectomy may pose a disproportionate risk to ovarian tissue. These options are generally not mentioned in the ACOG or NICE guidelines, possibly reflecting a more conservative approach or regional practice variation [5,23]. Furthermore, ESGE/ESHRE/WES uniquely advocate for surgical caution at the ovarian hilus and recommend avoiding bipolar coagulation in this area, with technical nuances that reflect a more specialized level of surgical detail absent in broader guidelines such as NICE and ACOG [29,33]. In addition, sclerotherapy has been proposed as a minimally invasive alternative for selected patients; however, ESHRE 2022 clearly states that this approach is not routinely recommended due to limited evidence and potential risks of chemical peritonitis and cyst recurrence [20]. The results of this comparison are shown in Table 3.

#### 2.2.2. Ovarian Reserve Assessment and Surgical Considerations (AMH, AFC)

Only a few guidelines delve into the measurable impact of surgery on ovarian reserve [34,35]. ESHRE 2022 and S2k explicitly recommend preoperative AMH testing to guide decision-making, particularly in cases of bilateral disease or prior ovarian surgery. In this context, AMH assessment is primarily used to inform surgical strategy selection and patient counseling, rather than to contraindicate surgery per se [20,36]. WES aligns with this approach, emphasizing that the potential negative impact of cystectomy on ovarian reserve must be weighed against the benefits [37]. This is a crucial point: while cystectomy is generally superior in symptom control, multiple studies have shown a postoperative decline in AMH levels, especially when bipolar energy is used or when the endometrioma is adherent to the cortex [38,39]. These observations derive from observational and meta-analytic evidence and are acknowledged, but not uniformly formalized, within guideline recommendations. This concern is under-recognized in ACOG and NICE, where there is less emphasis on ovarian reserve metrics in surgical planning. Interestingly, ESHRE also notes that AMH levels may drop even in patients with unoperated endometriomas, complicating the interpretation and making baseline measurements even more relevant [40,41]. The results of this comparison are shown in Table 4.

#### 2.2.3. Timing of Endometrioma Surgery Relative to ART/IVF

This section focuses on guideline-specific nuances regarding ART timing, without reiterating general principles discussed above. In this context, a major paradigm shift in the ESHRE 2022 and S2k 2025 guidelines is the rejection of routine endometrioma excision prior to ART. Surgery is now not recommended prior to MAR unless there is a clinical indication, such as pain, technical difficulty in oocyte retrieval, or suspicion of malignancy. WES supports this position, promoting ART access without delay [42,43,44]. This view contrasts with past practices where surgery was often performed in anticipation of better IVF outcomes. ACOG and NICE do not provide clear guidance on this timing, although NICE does mention that surgery should not be used solely to improve fertility outcomes in asymptomatic women [45]. Thus, current guidelines discourage surgical delays to ART unless strongly indicated, reflecting an evidence-based shift toward conservative therapies and expedited access to treatment [46]. The results of this evaluation are shown in Table 5.

#### 2.2.4. Management of Bilateral Endometriomas

Bilateral endometriomas present a unique clinical challenge. All guidelines recognize the heightened risk of diminished ovarian reserve, especially with repeat surgery. ESHRE, S2k, and WES all emphasize the need for preoperative fertility counseling and, in some cases, consideration of oocyte or embryo cryopreservation prior to surgery [47,48]. ESHRE uniquely advises that in bilateral disease, surgery should only be undertaken when necessary and ideally by experienced surgeons. Ablative techniques may be preferred in this setting to preserve ovarian tissue [49]. NICE, again, does not provide specific guidance here, and ACOG does not address bilateral disease separately. The results of this evaluation are shown in Table 6.

### 2.3. Surgery for Superficial Peritoneal Endometriosis

International guidelines show marked differences in their recommendations regarding the optimal surgical approach for peritoneal endometriosis, particularly when comparing excision and ablation. Both the ESHRE 2022 and S2k 2025 guidelines generally favor excision of peritoneal lesions, citing greater efficacy in symptom relief and lower recurrence rates compared to ablation [36,50]. In contrast, the NICE guideline does not express a definitive preference, instead advising that the choice between excision and ablation be individualized based on lesion characteristics and surgical expertise [23]. The ACOG guidance acknowledges that while both techniques can be effective, excision may offer longer-lasting pain control in certain subgroups [5,51]. Pain severity and response to hormonal therapy remain key determinants for surgical referral across all documents. ESHRE recommends surgery primarily for women who experience persistent or severe symptoms despite appropriate medical treatment, emphasizing the importance of tailoring decisions to symptom burden rather than solely anatomical findings [20]. Similarly, the ACOG and German S2k guidelines highlight that medical therapy should precede surgery in most cases unless there are urgent indications such as hydronephrosis or bowel stenosis [52,53]. NICE, meanwhile, encourages reevaluation after 6–12 months of medical therapy before considering operative management [54]. With regard to pain outcomes, most guidelines report that surgical excision is associated with improved symptom control and quality of life in women with persistent or severe pain. In contrast, the impact of surgery on fertility outcomes is more heterogeneous, with guidelines differing in the strength of recommendations and in patient selection criteria. While ESHRE and S2k report a potential increase in spontaneous pregnancy rates after surgery, especially for Stage I–II disease, they also caution against surgery in women with advanced age or diminished ovarian reserve, where expedited ART may be more appropriate [55,56]. ACOG notes that the effect of surgery on fertility is limited and context-dependent, whereas NICE underlines the importance of shared decision-making, particularly when ART is anticipated [57]. Finally, ESHRE and NICE favor a conservative, stepwise approach, avoiding surgery before ART unless clinically indicated. In contrast, the S2k guideline supports earlier surgery in symptomatic women not pursuing immediate conception. The results of this comparison are shown in Table 7.

### 2.4. Surgery in Deep Infiltrating Endometriosis (DIE)

Surgical treatment for deep infiltrating endometriosis (DIE) is generally indicated when patients present with persistent pelvic pain, functional obstruction of pelvic organs such as bowel or ureters or infertility that does not respond to medical management [58]. According to the ESHRE guideline, primary reasons for surgery include severe pain, hydronephrosis, or failure of hormonal therapy. Similarly, the German S2k guideline underscores that surgical intervention becomes appropriate in cases of urinary or intestinal obstruction and in the presence of intense or progressive symptoms. A more cautious approach is advised by ACOG and NICE, which recommend surgery primarily when medical treatments fail and when the impact on quality of life becomes significant [59]. The disease most commonly affects the rectum, sigmoid colon, bladder, and ureters. Among the reviewed guidelines, the S2k stands out for providing specific management pathways for ureteral and bowel involvement, along with recommendations for interdisciplinary referral [60]. While ESHRE and WES also acknowledge the anatomical complexity of posterior compartment disease, their approach tends to be more general, focusing on principles rather than algorithms [20]. From a technical perspective, the surgical strategy should be tailored to disease severity and anatomical involvement. In this context, conservative disc excision techniques for low- and mid-rectal DIE, including combined laparoscopic–transanal approaches (e.g., Rouen technique), have been reported to achieve complete excision while preserving rectal anatomy and function, with favorable functional and quality-of-life outcomes [61]. ESHRE and S2k both outline a graduated approach: conservative excision or “shaving” for superficial lesions, discoid resection for isolated transmural nodules, and segmental bowel resection for multifocal or extensive disease. The preference is to choose the least aggressive option that is still effective, minimizing potential complications [62]. NICE does not indicate a clear preference among techniques but recommends that decisions be made based on the surgeon’s experience and the specifics of each case [63,64]. Multidisciplinary collaboration is considered essential, particularly in advanced cases. The S2k guideline strongly emphasizes the importance of joint management involving gynecologic surgeons, colorectal surgeons, and urologists, especially when urinary tract or bowel resection is required [65,66]. Both ESHRE and WES advocate for care within specialized endometriosis centers, particularly for surgeries involving ureteral reimplantation or colorectal procedures. In contrast, ACOG and NICE acknowledge the usefulness of a multidisciplinary team but do not mandate it. Overall, European guidelines tend to provide more detailed, procedure-oriented recommendations and explicitly promote centralized, multidisciplinary care, whereas non-European guidelines adopt a broader, principle-based approach with less procedural granularity. As for complications, European guidelines tend to be more specific. ESHRE and S2k clearly describe the risks associated with DIE surgery, including damage to the ureters, rectum, bladder, or pelvic nerves, as well as potential long-term bowel dysfunction. These risks are explicitly acknowledged in European guidelines and further supported by evidence from observational surgical series. These documents also emphasize the importance of surgical expertise to prevent such outcomes [67,68]. WES reinforces this point, highlighting the need for thorough preoperative imaging and adaptability during surgery. ACOG and NICE adopt a more general approach, recognizing that surgical morbidity exists but without detailing how it may vary depending on the site or technique employed [19]. The results of this comparison are shown in Table 8.

### 2.5. Endometriosis and Infertility: Surgery vs. Assisted Reproductive Technologies (ART)

The question of whether surgery should be performed before initiating assisted reproductive technologies (ART), or whether ART should be pursued directly without prior surgery, remains a central point of discussion in the management of endometriosis-related infertility [69,70]. Most international guidelines caution against performing surgery routinely in women who are asymptomatic, advocating instead for a case-by-case assessment based on the overall clinical picture. For instance, both the ESHRE guidelines and the updated German S2k recommendations suggest that surgery should be reserved for specific scenarios, such as intense pelvic pain, suspected malignancy, or when anatomical alterations might compromise access during oocyte retrieval [71,72,73]. The WES position is in line with this view, underscoring the need to facilitate timely ART access and avoid unnecessary surgical delays. NICE also advises against intervention in the absence of symptoms, clearly warning against postponing fertility treatment without strong clinical justification [74]. Among the most debated indications for pre-ART surgery is the presence of endometriomas, particularly those measuring over 3 cm. While not all guidelines agree on a strict threshold, both ESHRE and S2k recognize that larger endometriomas—especially if symptomatic or obstructing access to ovarian follicles—may warrant surgical removal [75]. NICE refers to the 3 cm size as a consideration, though it stops short of designating it as a formal indication. Conversely, SEUD recommends a more conservative stance, advising against routine excision regardless of cyst size unless function or symptoms are significantly impacted [76,77]. Another complex scenario arises when deep infiltrating endometriosis (DIE) is accompanied by tubal occlusion. In these situations, ESHRE supports surgical intervention when it may improve ART outcomes or restore pelvic anatomy relevant to conception. The S2k guideline echoes this by suggesting that addressing both symptoms and mechanical infertility through surgery can be beneficial. ACOG and NICE are more reserved, offering limited guidance on the intersection between DIE and tubal factor infertility, and instead maintaining a focus on symptom severity as the primary driver of treatment [78,79]. In the broader debate between a “surgery-first” or “ART-first” approach, current evidence favors tailoring the decision to the patient’s individual circumstances. ESHRE, S2k, and WES tend to recommend prioritizing ART in women with low ovarian reserve or minimal symptoms, to avoid the additional risk of compromising ovarian function through surgery [70]. However, for younger patients with stable ovarian reserve and a preference for spontaneous conception, surgery may still be an appropriate first step [80,81]. NICE and ACOG avoid endorsing a single approach, emphasizing instead the importance of shared decision-making and a personalized care plan that reflects both clinical priorities and patient preferences [82]. Special considerations: adenomyosis and deep infiltrating endometriosis (DIE). In women with adenomyosis and infertility, guidelines generally do not recommend routine surgery prior to ART. Instead, they favor primary ART when reproductive timing or ovarian reserve is a concern, with consideration of short pre-ART medical suppression (e.g., GnRH analogues) on a case-by-case basis, given the limited quality of evidence [20] (ESHRE 2022; S2k 2025). For DIE, surgery should not be performed solely to enhance ART outcomes; primary ART is preferred in asymptomatic patients or when ovarian reserve is low. Surgery is reserved for significant pain unresponsive to medical therapy, organ compromise (e.g., hydronephrosis, bowel stenosis), or to facilitate safe oocyte retrieval when access is technically difficult. NICE similarly emphasizes individualized decision-making and avoiding delays to ART in sub-fertile patients unless clear clinical indications for surgery exist [23]. The results of this comparison are shown in Table 9.

### 2.6. Surgery for Recurrent Endometriosis

The decision to perform a second surgery for endometriosis should be made with great caution and individualized according to each patient’s symptoms, reproductive plans, and prior surgical history. Importantly, guideline recommendations implicitly differentiate between ovarian and non-ovarian recurrence, as the surgical risk profile and potential impact on fertility differ substantially between these scenarios. Both the ESHRE 2022 and S2k 2025 guidelines emphasize that repeat surgery should only be considered in cases of persistent or recurrent pain such as dyspareunia, pelvic pain, or bowel/bladder dysfunction that significantly impair quality of life or daily functioning [83]. In non-ovarian recurrence, particularly in deep infiltrating endometriosis, reoperation is primarily driven by symptom severity or organ dysfunction rather than fertility considerations. Before any reoperation, a comprehensive assessment of the risk–benefit balance is essential, taking into account factors such as the risk of nerve injury, adhesion formation, and potential reduction in ovarian reserve [84,85]. This concern is particularly relevant in cases of recurrent ovarian endometriomas, where repeat cystectomy is consistently associated with a higher risk of cumulative ovarian damage and reduced ovarian reserve. In many instances, continued medical management represents a reasonable alternative to reintervention, especially for women who have completed childbearing or have milder symptoms [83]. Hormonal suppression with progestins, combined oral contraceptives, or GnRH analogues is endorsed by all major guidelines as an effective option for controlling symptoms and potentially delaying or avoiding further surgery [86]. Regarding outcomes, guidelines note that while reoperation can offer symptom relief and functional improvement in selected cases, the predictability of success is lower than after primary surgery. All guidelines emphasize that cumulative surgical risk increases with each subsequent procedure, reinforcing the need for careful patient selection and long-term treatment planning. This is particularly true in patients with ovarian endometriomas, extensive adhesions, or bowel involvement, where recurrence risk and surgical complexity are higher [87]. Improvements in quality of life have been documented in some women, but these benefits tend to be less consistent compared with outcomes after first-line surgical management [85,88].

### 2.7. Radicality of the Surgery, How Far Should We Go?

The determination of whether to pursue conservative or radical surgical intervention for endometriosis requires careful individualization, considering reproductive goals, symptom severity, and response to prior medical or conservative surgical treatments. In this review, the term radical surgery refers to extensive excisional procedures that may include bowel or urinary tract resection, whereas definitive surgery is used specifically to indicate hysterectomy with or without oophorectomy. Within this context, guidelines clearly distinguish between hysterectomy as a definitive option for symptom control in women who have completed childbearing, and organ-sparing radical surgery aimed at complete excision of disease while preserving reproductive organs. ESHRE 2022 and S2k 2025 recommend considering hysterectomy with or without oophorectomy only for women who have completed childbearing and continue to experience severe, treatment-resistant symptoms that significantly impair quality of life, despite optimal medical therapy. These procedures are described as definitive surgical options, to be performed only after detailed counseling regarding their irreversible nature and potential long-term consequences [89]. Both guidelines acknowledge that radical surgery can provide substantial pain relief and improved quality of life in appropriately selected patients, but they emphasize that the procedure should not be considered a universal solution. Instead, it should follow a stepwise approach prioritizing conservative management first, a principle also shared in other uterine conditions in fertile age [90]. The removal of ovaries must be particularly well justified, given the associated risks of earlier menopause, bone loss, cardiovascular complications, and sexual dysfunction, which are consistently highlighted across international recommendations [91]. NICE 2024 places particular emphasis on shared decision-making, recommending that choices about hysterectomy and oophorectomy reflect the patient’s preferences, comorbidities, and individual risk tolerance. ACOG similarly recognizes hysterectomy as an appropriate option in cases of adenomyosis or chronic pelvic pain unresponsive to medical therapy, but does not recommend routine oophorectomy. Finally, the German S2k guideline broadens the concept of surgical radicality beyond hysterectomy, also encompassing bowel and urinary tract resections in deep endometriosis. It specifies that the extent of resection whether shaving, disc excision, or segmental resection should be determined according to lesion depth, organ involvement, and symptomatology, ideally within a multidisciplinary setting.

### 2.8. Integration Between Surgery and Pharmacologic Treatment

Postoperative hormonal therapy is consistently recommended across major international guidelines as an effective strategy to reduce recurrence and control pain after conservative surgery for endometriosis. In particular, ESHRE 2022, S2k 2025, and NICE 2024 recommend postoperative hormonal suppression in women not seeking immediate pregnancy, most commonly using continuous combined oral contraceptives (COCs), progestins (including dienogest), and/or the levonorgestrel-releasing intrauterine system (LNG-IUS); GnRH agonists are generally reserved for selected cases, usually in combination with add-back therapy. ACOG and WES also acknowledge the role of postoperative hormonal suppression in reducing symptom recurrence, although they provide less detailed guidance regarding specific regimens and duration. Overall, European guidelines emphasize long-term hormonal suppression as a key strategy for sustained symptom control and recurrence reduction following conservative surgery. ESHRE and NICE emphasize that continuous postoperative suppression is associated with a meaningful reduction in symptom and lesion recurrence after conservative surgery, particularly with sustained adherence [20,83]. The guidelines also note that the choice of hormonal agent should be individualized according to patient tolerance, side-effect profile, and reproductive goals. LNG-IUS and dienogest are often highlighted as preferred options because of their favorable efficacy and tolerability profile, findings that are supported by pooled evidence showing significant reductions in recurrence and pain scores [92]. In contrast, preoperative hormonal therapy is not recommended by any of the analyzed guidelines, as it does not appear to improve surgical outcomes or reduce intraoperative complexity. Instead, medical therapy is reserved for postoperative maintenance or for patients who decline or are not candidates for surgery [20].

### 2.9. Surgery and Quality of Life

All major international guidelines emphasize that surgical success in endometriosis should not be assessed solely by the completeness of lesion excision, but rather by improvements in patient-reported outcome measures (PROMs). In particular, ESHRE 2022 and S2k 2025 explicitly recommend the use of validated PROMs such as the Endometriosis Health Profile-30 (EHP-30), SF-36, and EQ-5D, while NICE 2024 encourages routine documentation of patient-reported pain and functional outcomes without mandating specific instruments. The ESHRE 2022 and S2k 2025 guidelines explicitly recommend the integration of validated PROMs—including the Endometriosis Health Profile-30 (EHP-30), SF-36, and EQ-5D—to assess treatment effectiveness and guide long-term follow-up. Similarly, NICE 2024 encourages clinicians to document patient-reported improvements in pain, sexual, urinary, and bowel function as part of outcome evaluation, aligning surgical goals with the patient’s perceived well-being. Evidence from structured reviews supports these guideline recommendations, confirming that validated PROMs capture meaningful improvements in pain, daily functioning, and sexual health following conservative laparoscopic surgery [20]. Improvements in pain scores and quality-of-life measures have been reported following conservative laparoscopic surgery; however, these findings derive mainly from observational studies and heterogeneous cohorts, and should be interpreted in light of study design, follow-up duration, and baseline symptom severity [93]. Accordingly, guidelines emphasize PROMs as complementary tools for outcome assessment rather than as isolated quantitative endpoints.

## 3. Conclusions

Across contemporary international guidelines (ESHRE 2022, S2k 2025, NICE 2024, WES, and ACOG), a consistent trend emerges toward individualized, symptom-driven, and fertility-oriented surgical management of endometriosis. Routine surgery in asymptomatic women—particularly before assisted reproductive technologies (ART)—is uniformly discouraged. Instead, all major societies emphasize shared decision-making, multidisciplinary expertise, and long-term postoperative hormonal therapy as central pillars to reduce recurrence and preserve quality of life. A critical aspect in comparing these documents is their publication age and evidence horizon. The ACOG 2010 guideline, despite its reaffirmation in 2022, is inherently based on data and clinical attitudes from more than a decade ago—an era preceding major advances in laparoscopic technology, fertility preservation, and ovarian reserve monitoring. In contrast, more recent guidelines such as ESHRE 2022, NICE 2024, and particularly the S2k 2025 represent an updated synthesis that integrates molecular insights, fertility outcomes, and patient-reported measures (PROMs) into surgical decision-making. This temporal evolution helps explain many of the observed discrepancies—older documents tend to adopt a more interventional or definitive stance, while newer ones reflect a conservative, evidence-based, and patient-centered philosophy. In conclusion, despite residual regional and temporal variations, global consensus is increasingly oriented toward conservative, fertility-preserving, and multidisciplinary surgical strategies. Future guideline revisions should aim to harmonize recommendations across societies, ensure timely updates to reflect emerging evidence, and maintain a strong focus on patient-centered outcomes as the primary benchmark of success in endometriosis surgery.

## Figures and Tables

**Table 1:** AGREE II-S domain scores and overall methodological quality of the included guidelines. ESHRE 2022 achieved the highest overall rating, reflecting strong evidence integration and editorial independence. In contrast, ACOG 2010, despite reaffirmation in 2022, showed the lowest score due to limited methodological transparency and an outdated evidence base.

Guideline	Scope and Purpose	Stakeholder Involvement	Rigor of Development	Clarity of Presentation	Applicability	Editorial Independence	Overall Quality (%)
**ESHRE 2022**	7	6	7	7	6	7	94
**S2k 2025**	7	6	6	7	6	6	90
**NICE 2024**	7	7	6	7	5	7	88
**SEUD 2023**	6	5	5	6	4	5	75
**ACOG 2010 (reaffirmed 2022)**	5	4	3	5	3	4	55
**WES 2013**	6	5	4	6	4	5	68

**Table 2:** **Indications for surgical management of ovarian endometriomas across international guidelines.** The table compares whether surgery is recommended only in symptomatic patients, presence of an explicit size cut-off (>3 cm), consideration of fertility impact, discouragement of surgery in asymptomatic women, and malignancy suspicion as an indication across six major guidelines (ESHRE 2022, S2k 2025, NICE 2024, SEUD 2023, ACOG 2010, WES 2013). This is a summary of recommendations from international guidelines regarding indications for surgical management of ovarian endometriomas.

Guideline	Surgery Only When Symptomatic?	Explicit Size Cut-Off (>3 cm)?	Mentions Impact on Fertility (ART)?	Surgery Discouraged If Asymptomatic?	Malignancy as Indication?
**ESHRE 2022**	Yes	No	Yes	Yes	Yes
**S2k 2025**	Yes	Yes	Yes	Yes	Yes
**NICE 2024**	Yes	No (mentions 3 cm without cut-off)	Yes	Yes	Yes
**SEUD 2023**	Yes	No	Yes	Yes	No (not specified)
**ACOG 2010**	Yes	No	Yes	No explicit statement	Yes
**WES 2013**	Yes	No	Yes	Yes	Yes

**Table 3:** Surgical techniques for ovarian endometriomas and fertility-preserving considerations across international guidelines. International guideline recommendations on surgical techniques for ovarian endometriomas. The table summarizes the preferred surgical technique (cystectomy), mentions of alternative technique modulation (e.g., laser ablation), specific cautions regarding ovarian hilum and bipolar energy use, and considerations for fertility preservation across major guidelines (ESHRE 2022, S2k 2025, ESGE/ESHRE/WES 2017, WES 2013, NICE 2024, ACOG 2010).

Guideline	Preferred Technique	Mentions Technique Modulation (e.g., Ablation)	Cautions on Ovarian Hilum/Bipolar Use	Notes on Fertility Preservation
**ESHRE 2022**	Cystectomy or laser ablation (both acceptable)	Yes—recommends switching to ablation if excision is difficult or may harm ovarian reserve	Yes	AMH testing; fertility-first approach
**S2k 2025**	Cystectomy	Yes—ablation allowed in high fertility risk or complex cases	Yes	Cryopreservation advised
**ESGE/ESHRE/WES 2017**	Cystectomy	Yes—recommends ablative approach when excision is technically difficult or poses risk to ovarian reserve	Yes	Specialist-level recommendations
**WES 2013**	Cystectomy	Implied—complex cases	No	Cortex preservation emphasized
**NICE 2024**	Cystectomy	Yes—ART context	No	Mentioned generically in ART settings
**ACOG 2010**	Cystectomy	No	No	Not addressed

**Table 4:** Guideline positions on the role of AMH testing and ovarian reserve considerations in surgical planning for endometriomas. This table compares international guidelines regarding the recommendation of anti-Müllerian hormone (AMH) testing, acknowledgment of AMH decline after surgery, the impact of thermal energy on ovarian reserve, recognition of AMH decrease in unoperated endometriomas, and the integration of AMH values into surgical decision-making.

Guideline	Recommends AMH Testing	Mentions AMH Decline After Surgery	Highlights Impact of Thermal Energy on AMH	Notes AMH Drop in Unoperated Endometriomas	Considers AMH in Surgical Decision-Making
**ESHRE 2022**	Yes	Yes	Yes	Yes	Yes
**S2k 2025**	Yes	Yes	Yes	No	Yes
**NICE 2024**	No	No	No	No	No
**SEUD 2023**	Not specified	Not specified	Not specified	Not specified	Not specified
**ACOG 2010**	No	No	No	No	No
**WES 2013**	Yes	Yes	Yes	No	Yes

**Table 5:** Guideline recommendations on surgical timing in relation to assisted reproductive technologies (ART). The table summarizes whether routine surgery before ART is advised, emphasizes clinical indications for surgery, highlights if timely IVF access should not be delayed, and specifies the main clinical situations where surgery may be appropriate prior to ART.

Guideline	Routine Surgery Before ART?	Surgery Only If Clinically Indicated?	Mentions IVF Access Should Not Be Delayed	Clinical Indications for Surgery
**ESHRE 2022**	No	Yes	Yes	Pain, oocyte retrieval difficulties, suspicion of malignancy
**S2k 2025**	No	Yes	Yes	Pain, oocyte retrieval difficulties, malignancy
**WES 2013**	No	Yes	Yes	Pain, functional impairment
**NICE 2024**	Not recommended if asymptomatic	Yes	Yes (indirectly)	Pain, oocyte retrieval difficulties
**ACOG 2010**	Not clearly addressed	Implied	No	Symptom-based

**Table 6:** Guideline recommendations for surgical planning in bilateral endometriomas. This table summarizes how guidelines address the risk to ovarian reserve, the importance of preoperative fertility counselling, recommendations for oocyte or embryo cryopreservation, support for conservative or ablative approaches to preserve ovarian function, and the role of experienced surgeons in complex cases.

	Recognizes Risk to Ovarian Reserve	Recommends Preoperative Fertility Counseling	Mentions Oocyte/Embryo Cryopreservation	Supports Conservative/Ablative Approach	Recommends Experienced Surgeon
**ESHRE 2022**	Yes	Yes	Yes	Yes	Yes
**S2k 2025**	Yes	Yes	Yes	Optional	Yes
**WES 2013**	Yes	Yes	Yes	Yes	Implied
**NICE 2024**	Yes (general)	Not explicit	No	No guidance	No
**ACOG 2010**	Not specified	Not addressed	No	No	No

**Table 7:** Surgical approach in superficial peritoneal endometriosis: excision vs ablation and timing relative to ART. This table compares guideline positions on the preferred surgical technique (excision or ablation), indications based on pain severity and response to medical therapy, expected clinical and reproductive benefits, conservative versus surgical-first strategies, and recommendations regarding the timing of surgery in relation to assisted reproductive technologies (ART).

Guideline	Excision vs. Ablation	Indication Based on Pain/Therapy Response	Clinical/Reproductive Benefit	Conservative vs. Surgical-First Approach	Timing Relative to ART
**ESHRE 2022**	Favors excision	Yes—persistent or severe symptoms	Improves pain; possible fertility benefit	Conservative; avoid surgery before ART	Surgery only if indicated
**S2k 2025**	Favors excision	Yes—therapy failure or urgent cases	Improves pain; early surgery if not pursuing ART	Selective early surgery allowed	Surgery only if indicated
**WES 2013**	Not explicit	Not detailed	General benefit acknowledged	Aligns with conservative model	Avoid delays to ART
**NICE 2024**	No preference—individualized	Yes—re-evaluate after 6–12 months	Quality-of-life benefit; no clear fertility stance	Conservative/stepwise	Avoid surgery before ART if asymptomatic
**ACOG 2010**	Both valid—excision may last longer	Yes—after failed medical therapy	Limited fertility effect; pain relief noted	Surgery based on symptoms	Not clearly addressed

**Table 8:** Indications, surgical techniques, and multidisciplinary approach in deep infiltrating endometriosis (DIE). The table summarizes guideline recommendations regarding indications for surgery in DIE, targeted anatomical sites, preferred surgical techniques (shaving, discoid or segmental resection), the role of multidisciplinary teams, and reported complications related to urinary tract, bowel, and nerve structures.

Guideline	Indications for Surgery	Anatomical Targets	Surgical Techniques	Multidisciplinary Approach	Complications Mentioned
**ESHRE 2022**	Severe pain, hydronephrosis, failure of hormonal therapy	Rectum, sigmoid colon, bladder, ureters	Shaving, discoid resection, segmental resection	Recommended in specialized centers	Detailed: ureter, rectum, bladder, nerves, bowel dysfunction
**S2k 2025**	Urinary or bowel obstruction, severe or progressive symptoms	Rectum, sigmoid colon, bladder, ureters	Shaving, discoid resection, segmental resection	Strongly recommended (gynecologist, colorectal surgeon, urologist)	Detailed: ureter, rectum, bladder, nerves, bowel dysfunction
**WES 2013**	Persistent symptoms, anatomical complexity	Posterior compartment (general)	General principles; less algorithmic	Advocated in specialized centers	Emphasizes imaging and intraoperative flexibility
**NICE 2024**	Failure of medical treatment, impact on quality of life	Rectum, sigmoid colon, bladder, ureters	No specific preference; based on surgeon experience	Acknowledged but not mandated	General reference only
**ACOG 2010**	Failure of medical treatment, impact on quality of life	Rectum, sigmoid colon, bladder, ureters	Not specified	Acknowledged but not mandated	General reference only

**Table 9:** Guidelines on surgery timing before ART and shared decision-making. Comparison of recommendations on when to perform surgery in relation to ART, considering endometrioma size, combined tubal involvement, ovarian reserve, and patient preferences.

Guideline	Routine Surgery Before ART?	Recognizes Endometrioma > 3 cm	DIE + Tubal Occlusion: Surgery Supported?	Surgery First in Young Women with Good Reserve?	ART First in Low Reserve/Asymptomatic?	Shared Decision-Making Emphasized?
**ESHRE 2022**	No	No formal cut-off	Yes	Yes (if symptoms or patient preference)	Yes	Yes
**S2k 2025**	No	Yes	Yes	Yes (if symptoms or patient preference)	Yes	Yes
**WES 2013**	No	Not stated	Not specified	Yes	Yes	Yes
**NICE 2024**	Not recommended if asymptomatic	Considered, not formalized	No clear guidance	Shared decision	Yes	Yes
**ACOG 2010**	Implied no	Not stated	No clear guidance	Shared decision	Yes	Yes

## Data Availability

The data that support the findings of this study are not publicly available but access can be requested from the authors via email.

## Abbreviations

ACOG	American College of Obstetricians and Gynaecologists
AGREE II	Appraisal of Guidelines for Research & Evaluation II
ART	Assisted Reproductive Technology
DIE	Deep Infiltrating Endometriosis
ESHRE	European Society of Human Reproduction and Embryology
IVF	In Vitro Fertilization
MDT	Multidisciplinary Team
NICE	National Institute for Health and Care Excellence
PROMs	Patient-Reported Outcome Measures
QoL	Quality of Life
SEUD	Society of Endometriosis and Uterine Disorders
S2k	S2k Guideline of the German Society of Gynecology and Obstetrics
VAS	Visual Analogue Scale
WES	World Endometriosis Society
